# Therapeutic prospects of bright light therapy in addictive disorders: a scoping review

**DOI:** 10.1007/s00702-026-03131-1

**Published:** 2026-03-17

**Authors:** Benjamin Rolland, Imane Salihi, Sibylle Mauries, Ilona Medigue, Sébastien Catoire, Laure Peter-Derex, Pierre Alexis Geoffroy

**Affiliations:** 1 Service Universitaire d’Addictologie de Lyon (SUAL), Hospices Civils de Lyon, Lyon, France; 2 Service Universitaire d’Addictologie de Lyon (SUAL), CH Le Vinatier, Bron, France; 3 Centre de Recherche en Neurosciences de Lyon (CRNL), UCBL1, INSERM 1028, CNRS, UMR 5292, Bron, France; 4 Département de psychiatrie et d’addictologie, GHU Paris Nord, DMU Neurosciences, AP-HP, Hôpital Bichat - Claude Bernard, Paris, F-75018 France; 5 Université Paris Cité, NeuroDiderot, Inserm U1141, Paris, F-75019 France; 6 GHU Paris - Psychiatrie & Neurosciences, Centre ChronoS, 1 rue Cabanis, Paris, 75014 France; 7 Service Universitaire des Pathologiques Psychiatriques Résistantes, Unité du Sommeil Michel Jouvet, CH Le Vinatier, Bron, France; 8 Centre de Médecine du Sommeil, Hospices Civils de Lyon, Lyon, France

**Keywords:** Bright light therapy, Substance use disorders, Sleep, Chronobiology, Behavioral addictions, Depression

## Abstract

Bright light therapy (BLT) is now established as an evidence-based treatment for several psychiatric and sleep disorders, with robust efficacy in seasonal affective disorder, non-seasonal, unipolar, and bipolar depression, insomnia and circadian rhythm sleep–wake disorders, including Delayed Sleep–Wake Phase Disorder. BLT-related mechanisms are multifactorial, combining circadian phase-shifting effects, stabilization of the sleep–wake cycle, improved homeostatic sleep pressure, and modulation of monoaminergic pathways. Beyond these effects, recent translational studies suggest that BLT also modulates the brain reward system through circadian-dopaminergic interactions, providing a neurobiological rationale for its use in disorders where motivation, reward sensitivity, and compulsive behaviors are disrupted. Despite these advances, BLT has been largely neglected in the field of addictive disorders, even though circadian disruption, sleep disturbances, and mood instability are central clinical features of addiction. This scoping review explores the therapeutic prospects of BLT in addictive disorders. We first summarize the established evidence supporting BLT for mood and sleep disorders, which provides a strong translational basis. We then discuss three potential applications in addiction: (1) alleviating comorbid or subthreshold mood and anxiety symptoms, which are highly prevalent and disabling in substance use and behavioral addictions; (2) improving sleep and circadian regulation, frequently impaired in these populations and closely linked to relapse vulnerability; and (3) directly modulating the reward system and core addictive behaviors. In conclusion, BLT offers many potential benefits for patients with addictive disorders. Well-designed trials are now needed to confirm efficacy, refine protocols, and integrate BLT into addiction care.

## Introduction

Daylight is a key modulator of human circadian and biological rhythms, with effects primarily mediated by the retina and intrinsically photosensitive retinal ganglion cells (ipRGCs) expressing melanopsin (Newman et al. 2003; Maruani and Geoffroy 2022). These cells detect short-wavelength light, corresponding to the blue region of the spectrum, and transmit signals to the suprachiasmatic nucleus (SCN) and other brain structures, thereby synchronizing the internal clock with external solar time (Wahl et al. 2019). Through this mechanism, sleep, mood, and other physiological and behavioral cycles are regulated (Hatori and Panda 2010; Dijk and Archer 2009). Beyond circadian entrainment, ipRGCs also influence mood, arousal, eating behavior and cognition via direct projections to limbic and thalamic regions, an increasingly recognized mechanism for the therapeutic effects of light (Maruani and Geoffroy 2022; Wahl et al. 2019; Hatori and Panda 2010; Dijk and Archer 2009; Lupi et al. 2008; Fleur et al. 2024; Montaruli et al. 2021).

For more than forty years, devices mimicking daylight have thus been developed to use light as a medical treatment for different categories of brain disorders. Bright Light Therapy (BLT) involves the exposure to a lightbox emitting 10,000 lx of full-spectrum visible light, typically conducted in the morning for optimal effectiveness (Campbell et al. 2017). Depending on the device, the lightbox should be placed 40–80 cm away, with the light meeting the eyes at a 30–60° angle, as direct staring is unnecessary. A standard daily starting dose is 30 min per session at 10,000 lx, though duration and intensity may be adjusted based on individual response. BLT is non-invasive, and provides a safe, drug-free alternative, for patients (pregnant women and children) who cannot tolerate or prefer to avoid pharmacological treatments (Campbell et al. 2017). So far, BLT has become a first-line intervention in several psychiatric and sleep disorders. Its efficacy has been best demonstrated in seasonal affective disorder, but it has also been extended to non-seasonal depression and bipolar depression, with structured recommendations now available (Geoffroy et al. 2025).

Despite these advances, BLT remains largely unexplored in the field of addiction, even though circadian disruption, sleep impairments, and mood instability are core features of addictive disorders (Medigue et al. 2025; Mauries et al. 2025; Catoire et al. 2021; Geoffroy et al. 2020; Meyrel et al. 2020). This is surprising as daylight exposure could have multiple beneficial effects on both substance use disorders (SUDs) and behavioral addictions. First, BLT may alleviate the burden of psychiatric disorders and sleep disorders that are frequently found comorbid with addictive disorders. Moreover, BLT could help reduce the cortege of psychiatric or sleep symptoms surrounding addictive disorders, even when no characterized psychiatric or sleep disorder is met. Finally, BLT may directly reduce the core symptoms of addictive disorders, which are strongly influenced by circadian rhythms through the inner clock and by the sleep-wake system. This narrative review aims to explore all these different yet complementary therapeutic prospects, after a brief overview of the current evidence regarding psychiatric indications of BLT.

## Bright light therapy: current evidence and main indications

### BLT for mood disorders

Though the use of light in medicine is very old, one of the earliest uses of BLT device in the psychiatric field was seasonal affective disorder (SAD). SAD is characterized by recurrent episodes of major depression, following a seasonal pattern—typically depression during fall and winter, with full remission in spring and summer (Munir et al. 2025). Since melatonin is involved in the regulation of seasonal rhythms, BLT has initially emerged as an obvious treatment for SAD, and a first study in this regard was published in 1984 (Rosenthal et al. 1984). Since then, numerous clinical trials and meta-analyses have confirmed the efficacy of BLT as a first-line treatment for SAD (Pjrek et al. 2020).

Subsequently, BLT has been progressively used and studied in nonseasonal depression, especially in major depressive disorder (MDD) (Geoffroy and Palagini 2021). Indeed, patients with nonseasonal depression frequently exhibit symptoms linked to circadian abnormalities, including irregular sleep-wake patterns and altered mood rhythms (Allen et al. 1993; Lieverse et al. 2011). Interestingly, BLT not only alleviates depressive symptoms but also improves cognitive functions associated with mood, such as optimism and decision-making (Sharot et al. 2007; Geoffroy et al. 2015; Praschak-Rieder et al. 1997). Moreover, BLT effect has a rapid onset, with clinical improvements typically observed within a week after starting therapy, which is much faster than all first-line antidepressants (Boyce and Hopwood 2013). Importantly, BLT has demonstrated clear efficacy in patients with moderate to severe major depressive episodes, as confirmed by a meta-analysis comparing light therapy, antidepressants, and their combination (Boyce and Hopwood 2013). This meta-analysis showed that BLT alone was as effective as antidepressant drugs, while the combination of BLT and antidepressants was superior to antidepressants alone, both in seasonal and non-seasonal depression (Geoffroy et al. 2019). Building on these findings, BLT has also shown encouraging preliminary results in moderate depression (Legenbauer et al. 2024). By contrast, the efficacy and especially the risk/benefit ratio of antidepressants in milder forms of depression remain debated, as available evidence is limited and such presentations are often less specific of a MDD (Fournier et al. 2010). In this context, BLT may represent an interesting and well-tolerated therapeutic option for subthreshold or mild-to-moderate depressive symptoms (Jiang et al. 2021), although further comparative studies are needed to clarify its relative benefit and optimal indications.

More recently, BLT has been recognized as an effective and safe treatment option for bipolar depression. The first institutional positioning came from the ISBD Chronobiology and Chronotherapy Task Force review, which emphasized the strong potential of chronotherapeutics and highlighted BLT as one of the most promising non-pharmacological approaches for bipolar disorder (Gottlieb et al. 2019). Building on this, the recent ISBD clinical recommendations further consolidated BLT as a first-line adjunctive treatment for bipolar depression, provided that timing, duration, and safety monitoring are carefully respected. Importantly, a recent randomized controlled trial showed that while BLT is efficacious, clinicians must remain attentive to the risk of mood switching. To reduce this risk, BLT should always be prescribed in association with a mood stabilizer, and, in patients considered at high risk for switch, a mid-day exposure protocol may be preferred over early-morning exposure. It is also recommended to carefully monitor early warning signs, such as emerging irritability, decreased need for sleep, or accelerated speech. In such cases, prompt clinical adjustment (e.g., temporary suspension or dose/timing modification) can help ensure safety (Geoffroy et al. 2025). Altogether, these data support the integration of BLT into treatment algorithms for bipolar depression, while underscoring the need for structured monitoring and individualized protocols.

The exact mechanisms of action of BLT in mood disorders remain incompletely understood, though they are likely to involve a combination of circadian and non-circadian effects. On the circadian side, BLT induces phase shifts of circadian rhythms, thereby resynchronizing the internal clock with the external light–dark cycle, which is particularly relevant in patients with mood disorders who often present with delayed or misaligned rhythms (Maruani and Geoffroy 2019; Pail et al. 2011). BLT also promotes alertness and stabilizes the sleep–wake cycle by acting on both SCN and extra-SCN pathways, contributing to improved daytime dysfunctions (Geoffroy et al. 2025; Maruani and Geoffroy 2019). In parallel, BLT influences sleep homeostasis by increasing slow-wave activity (delta EEG power) and sleep pressure, which may help restore restorative sleep and reduce depressive symptoms. Non-circadian mechanisms also play a critical role. BLT modulates serotoninergic, dopaminergic, and other monoaminergic pathways, consistent with its rapid antidepressant effects compared with pharmacological treatments (Maruani and Geoffroy 2022; Geoffroy and Palagini 2021). Recent translational work has emphasized the role of iPRGCs and their melanopsin-driven projections to mood-related brain regions, such as the lateral habenula and limbic structures, beyond the classical circadian pacemaker (Maruani and Geoffroy 2022). These multiple pathways, dependent and independent of SCN pathways, are not mutually exclusive and likely converge to produce the clinical effects of BLT (Maruani and Geoffroy 2022; Geoffroy et al. 2025).

## BLT for sleep disorders

BLT gained a prominent position among treatments for sleep disorders. Indeed, it is now listed among high evidence based-level adjunctive options in the 2023 European Insomnia Guidelines (Riemann et al. 2023), and increasingly studied and recommended for circadian rhythm disorders, notably delayed sleep–wake phase syndrome, and hypersomnia and related disorders.

First, BLT is a cornerstone therapy for Delayed Sleep–Wake Phase Disorder (DSWPD). Early clinical work showed that morning bright light advances circadian phase and improves sleep timing in DSWPD (Auger et al. 2015; Dijk et al. 2012; Gronfier et al. 2004). BLT has demonstrated efficacy particularly in intrinsic DSWPD, characterized by a biologically confirmed circadian phase delay, and in behaviorally reinforced delayed sleep phase frequently observed in addictive disorders, leading to circadian phase advance, earlier sleep timing, improved sleep continuity, and better daytime functioning (Geoffroy et al. 2025; Faulkner et al. 2019). The current American Academy of Sleep Medicine (AASM) recommendations emphasize tailoring light exposure to the phase response curve: morning light to advance the circadian phase, evening light to delay it, often combined with behavioral anchoring strategies such as fixed wake times (Auger et al. 2015). Where appropriate, timed melatonin supplementation is recommended as an adjunct. These approaches are grounded in decades of chronobiology research. Recent systematic reviews and consensus papers further synthesize these effects, offering clinicians pragmatic algorithms for the evaluation and treatment of intrinsic and extrinsic circadian rhythm sleep–wake disorders (e.g., delayed sleep–wake phase disorder, non-24-h sleep–wake disorder, shift work disorder). By aligning therapy with the circadian system’s physiology, clinicians can significantly improve the outcomes of patients with psychiatric and non-psychiatric comorbidities (Geoffroy et al. 2025; Faulkner et al. 2019).

For insomnia disorder (chronic insomnia), BLT is not a replacement for CBT-I, but growing evidence supports its adjunctive value, with a high-level of evidence, particularly for sleep maintenance problems and circadian misalignment (Riemann et al. 2023; Auger et al. 2015; Dijk et al. 2012; Gronfier et al. 2004; Faulkner et al. 2019; Baglioni et al. 2020). In a systematic review and meta-analysis focused on insomnia disorder, BLT reduced wake after sleep onset (WASO) measured objectively and subjectively (Chambe et al. 2023). BLT thus seems to show modest but positive effects on sleep quality in insomnia, while calling for higher-quality randomized clinical trials (RCTs) to refine spectral dose, duration, and timing by phenotype.

Furthermore, BLT shows growing interest in treating hypersomnia and excessive daytime sleepiness (EDS) (Souman et al. 2018). While clinical evidence specific to idiopathic hypersomnia or narcolepsy remains limited, BLT’s alerting effect and its ability to stabilize circadian phase support its use as an adjunct in patients with EDS associated with sleep deprivation (Comtet et al. 2019), neurological conditions (Raikes et al. 2020; Videnovic et al. 2017), and sleep disorders such as circadian rhythm disturbances or obstructive sleep apnea (Soreca et al. 2024). BLT appears also to improve daytime vigilance and rhythm amplitude when morning exposure is applied. Beyond daytime sleepiness, some evidence suggests that sleep inertia following nighttime awakening may benefit from light exposure, although large RCTs are still lacking (Hilditch et al. 2022; Didikoglu et al. 2023).

Across these circadian and sleep indications, BLT appears well-tolerated, with most common side effects being transient headache and eye strain. Typical clinical parameters are similar to those used for depression, with daily exposure to 10,000 lx (or equivalent if using blue-enriched light) for 30 min. Morning exposure is recommended to induce a phase advance (in case of DSWPD and most insomnia phenotypes), whereas evening exposure is used to produce a phase delay when indicated (less often), and early morning or nighttime exposure may be employed to transiently increase alertness. Sleep education and behavioral interventions should be associated, including a fixed wake-up time and appropriate light-dark hygiene.

## Other neuropsychiatric indications of BLT

BLT has been shown to effectively reduce satellite symptoms of anxiety and depression in different types of chronic diseases or disorders, even in patients with no characterized psychiatric disorder. For example, in a randomized controlled trial involving 101 participants with medically intractable epilepsy, those who received 12 weeks of daily light therapy (either high or low intensity) showed significant improvements in anxiety and depression scores (Baxendale et al. 2013). There were no significant differences between the high- and low-intensity groups in terms of effectiveness, suggesting that even lower intensity light therapy could be beneficial. The reduction in anxiety and depression was unrelated to seizure frequency, emphasizing that mood improvements were likely due to the therapeutic effects of BLT rather than changes in seizure activity. Similarly, in a sample of 125 adults with non-specific chronic back pain, BLT, even in low dose, could improve global depressive symptoms and reduce pain intensity, even if the study duration did not exceed three weeks (Elicit et al. 2014). In dementia, BLT was found associated with improved mood, more efficient sleep, and enhanced stimulation in a sample of institutionalized older adults with moderate to very severe dementia (Riemersma-van der Lek et al. 2008).

BLT has been investigated in other psychiatric disorders beyond depression and sleep disorders, e.g., post-traumatic stress disorder (Millot et al. 2024), eating disorders (Beauchamp and Lundgren 2016), or borderline personality disorder (Prasko et al. 2010), but data remain extremely limited at this stage. Though BLT has not been properly assessed in patients with characterized anxiety disorders, such as generalized anxiety disorder or panic disorder, the effect of BLT on anxiety symptoms has been investigated in several pilot trials, with small or negative efficacy (Youngstedt and Kripke 2007). Finally, BLT has shown some interesting preliminary results for treating negative symptoms of schizophrenia, which are often resistant to standard antipsychotic treatments (Aichhorn et al. 2007; Roopram et al. 2016).

## Potential applications of bright light therapy in addictive disorders

**Fig. 1:**
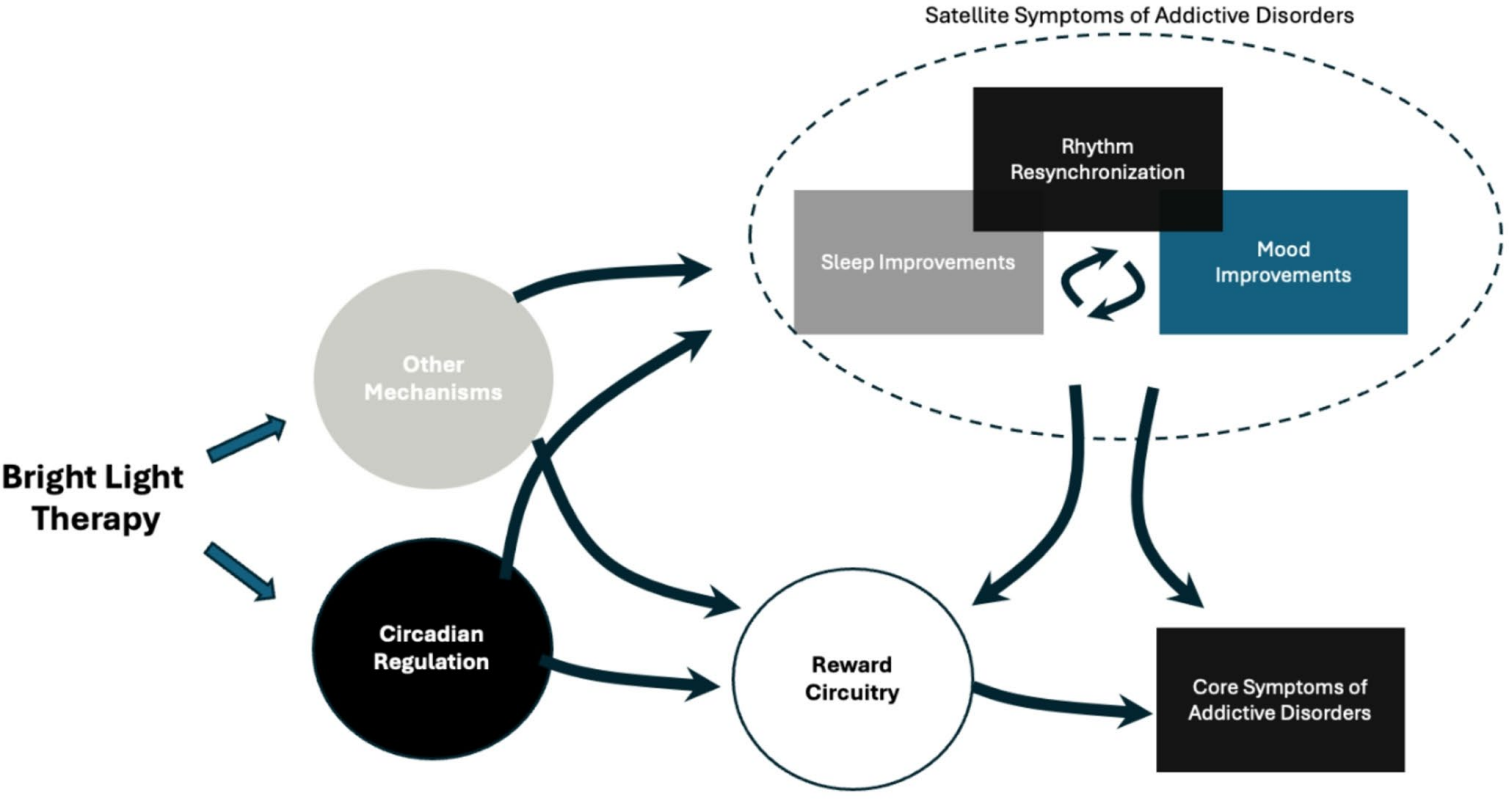
Potential mechanisms of action of bright light therapy in addictive disorders

Different neurobiological pathways can support the interest of using BLT in addictive disorders (see Fig. 1). Given the current available evidence, BLT appears as a good candidate for alleviating comorbid mood or sleep impairments that are found frequently associated with addictive disorders. As previously seen, these effects can be mediated by in improvements in the functioning of circadian centers, or be based on other mechanisms. Indirectly, such improvements could also lead to alleviating the core symptoms of addiction, including craving, loss of control, and thus have an impact on behavioral outcomes related to addiction, e.g., frequency and level of use. In addition, BLT could directly affect the brain reward circuitry, thus leading to direct improvement of addiction symptomatology.

## BLT for treating satellite mood symptoms in addictive disorders

As previously mentioned, an increasing number of studies have found that BLT could improve depressive symptoms related to diverse chronic diseases or chronic disorders, even when no criteria for a characterized psychiatric disorder are found. The examples of using BLT for treating satellite mood or anxiety symptoms in epilepsy (Baxendale et al. 2013), non-specific chronic back pain (Elicit et al. 2014), or dementia (a majority of whom had Alzheimer disease or vascular dementia) (Riemersma-van der Lek et al. 2008) have been mentioned above. In addictive disorders, satellite mood and anxiety symptoms are highly prevalent, and often correspond to subthreshold symptoms causing significant disability and distress. Such symptoms have been found to frequently occur in SUDs (Johnson et al. 2022; Tiguman et al. 2022), where they can be both the cause and the consequence of addiction and/or direct effects of the underlying substance (Schuckit 1996). General anxiety and mood symptoms, sometimes leading to characterized anxiety and mood disorders, are also frequent in behavioral addictions (Starcevic and Khazaal 2017; Alavi et al. 2012).

As previously demonstrated in other chronic diseases, BLT could be a safe and effective first-line treatment to reduce satellite mood, anxiety and sleep symptoms in addictive disorders, or an interesting add-on treatment to reduce characterized comorbid anxiety, mood and disorders in both SUDs and behavioral addictions. Feasibility studies should first determine whether the technical procedures should be aligned with those employed in similar studies, and more specifically, it is important to assess whether BLT use is more effective and acceptable in abstinent patients, i.e., those who have already stopped substance use, or whether BLT use is applicable in patients who continue to use large amounts of alcohol or drugs .

### BLT for treating satellite sleep impairments and comorbid sleep disorders

Sleep and circadian rhythms are particularly disrupted in patients with addictive disorders. Objective and/or subjective sleep disturbances have been found in diverse SUDs, e.g., tobacco (Catoire et al. 2021), alcohol (Reid-Varley et al. 2020), cannabis (Brown et al. 2024), cocaine (Medigue et al. 2025), opioids (Brown et al. 2024), as well as in gambling disorder (Austin et al. 2024), sexual addiction (Zwerling et al. 2021), or video gaming disorder (Christens et al. 2021). While each substance exerts a specific effect on sleep architecture (Gordon 2019), the global addiction process also deeply impairs sleep and wakefulness, probably by disrupting social habits and circadian rhythms (Tamura et al. 2021). Sleep impairments associated with addictive disorders have been poorly addressed in internal recommendations for treating SUDs and behavioral addictions, whereas improving sleep and wakefulness could yield multiple benefits (Geoffroy et al. 2020; Roncero et al. 2020). More specifically, restoring a better sleep quality, duration, and regularity, could improve the clinical outcomes of addictive disorders, that is, by reducing the level of substance use or addictive behaviors. Improving sleep can also contribute to reduce mood, anxiety and cognitive symptoms, and thus have an overlapping effect with the previous type of possible use of BLT in addictive disorders, i.e., for treating anxiety and mood symptoms. Finally, improving sleep could contribute to enhance general well-being and quality of life in patients with addictive disorders.

BLT appears as a promising strategy for reducing sleep impairments in patients with SUDs or with addictive disorders, given its previously demonstrated efficacy in many other conditions in which sleep is affected. If BLT is found to improve sleep in patients with addictive disorders, it will be important to assess whether this effect stems primarily from chronobiological realignment, or if it is independent of circadian synchronization. Most likely, these mechanisms are complementary, with circadian resynchronization, improved sleep continuity, and downstream effects on mood and anxiety synergistically contributing to enhance clinical outcomes. In this regard, BLT could offer a multi-layered benefit that targets several of the key vulnerabilities in addictive disorders.

## BLT for improving core outcomes of addictive disorders

Moreover, recent findings suggest that the reward circuitry and the circadian oscillators are deeply interconnected (Becker-Krail et al. 2022; Falcon et al. 2013), suggesting that BLT could alleviate the core symptoms of addictive disorders. In the treatment of addictive disorders, the main clinical outcomes generally pertain to decreasing the frequency and levels of substance use, or the frequency and levels of addictive behaviors (Narváez-Camargo et al. 2025) such as gambling, sex, or video gaming (Yau and Potenza 2015). Additional core outcomes consist of reducing the negative consequences related to substance use or addictive behaviors. These negative consequences can be very widespread and include both social and medical parameters. In practice, the DSM-5 criteria, currently the most widely used tool for measuring the severity of addictive disorders, encompass a broad range of pharmacological, psychological, behavioral, and social outcomes. To date, SUDs and behavioral addictions have been treated by a mix of pharmacotherapy and psychotherapy. In some disorders, approved medications are available, whereas in others, in particular in behavioral addictions, no medication has been approved, and pharmacotherapy use remains off-label or restricted to research protocols (Oliva et al. 2011). The use of medical devices for treating addictive disorders is relatively uncommon, with most applications relying on neurostimulation approaches, such as transcranial direct current stimulation or repetitive transcranial magnetic stimulation (Mehta et al. 2024).

The rationale for using BLT for improving the core outcomes of addictive disorders is less straightforward than that supporting the use of BLT for alleviating satellite mood, anxiety, or sleep disorders. Despite this, there are some arguments to support the use of BLT as a direct treatment of SUDs and behavioral addictions, the most important of which is that disrupted circadian rhythms have been suggested to play a central role in the pathophysiology of addiction (Sharma and Nelson 2024; Logan et al. 2014). The addiction process seems to deeply disrupt the internal clock, because of direct neurobiological effects, but also because addiction fragments the social and behavioral habits, that help to constantly adjust the internal clock with the outside reality (Sharma and Nelson 2024). Thus, aiming to resynchronize the internal clock and improve biological and social rhythms has been proposed as a central therapeutic option in the treatment of addictive disorders (Logan et al. 2014; Peyron et al. 2024). The strategy of using natural light for improving the clinical outcomes of addiction has been a longstanding strategy in natural-based approaches (Díaz-Martínez et al. 2024), even if such approaches mix the effects of light with other nature-related potential health benefits. In this respect, BLT appears as a more light-focused, more structured, and more reproducible therapeutic approach, which can be easier to assess in randomized controlled trials. Implementing BLT sessions in routine clinical practice seems particularly simple, especially if the session is performed in a hospital setting, for example in day hospital programs.

In conclusion, BLT is a simple, structured and validated approach for fostering circadian resynchronization, and for improving sleep and mood in different types of disorders. These effects should be definitively explored also in SUDs, and also with the aim to investigate whether BLT allows to improve the core symptoms and main outcome criteria of SUDs. In this respect, double-blind RCTs assessing the acceptability, efficacy, and tolerability of BLT are warranted in patients with SUDs.

## Data Availability

No datasets were generated or analysed during the current study.
